# Effect of solid-electrolyte pellet density on failure of solid-state batteries

**DOI:** 10.1038/s41467-024-45030-7

**Published:** 2024-01-29

**Authors:** Mouhamad S. Diallo, Tan Shi, Yaqian Zhang, Xinxing Peng, Imtiaz Shozib, Yan Wang, Lincoln J. Miara, Mary C. Scott, Qingsong Howard Tu, Gerbrand Ceder

**Affiliations:** 1 Department of Materials Science and Engineering, University of California, Berkeley, CA 94720 USA; 2 Department of Mechanical Engineering, Rochester Institute of Technology, Rochester, NY 14623 USA; 3 Advanced Materials Lab, Samsung Advanced Institute of Technology-America, Samsung Semiconductor Inc., Cambridge, MA 02138 USA; 4 National Center for Electron Microscopy, Molecular Foundry, Lawrence Berkeley National Laboratory, 1 Cyclotron Road, Berkeley, CA 94720 USA; 5 Materials Sciences Division, Lawrence Berkeley National Laboratory, Berkeley, CA 94720 USA

**Keywords:** Batteries, Batteries

## Abstract

Despite the potentially higher energy density and improved safety of solid-state batteries (SSBs) relative to Li-ion batteries, failure due to Li-filament penetration of the solid electrolyte and subsequent short circuit remains a critical issue. Herein, we show that Li-filament growth is suppressed in solid-electrolyte pellets with a relative density beyond ~95%. Below this threshold value, however, the battery shorts more easily as the density increases due to faster Li-filament growth within the percolating pores in the pellet. The microstructural properties (e.g., pore size, connectivity, porosity, and tortuosity) of $75\%{{{{{\rm{L}}}}}}{{{{{{\rm{i}}}}}}}_{2}{{{{{\rm{S}}}}}}-25\%{{{{{{\rm{P}}}}}}}_{2}{{{{{{\rm{S}}}}}}}_{5}$ with various relative densities are quantified using focused ion beam–scanning electron microscopy tomography and permeability tests. Furthermore, modeling results provide details on the Li-filament growth inside pores ranging from 0.2 to 2 μm in size. Our findings improve the understanding of the failure modes of SSBs and provide guidelines for the design of dendrite-free SSBs.

## Abstract (web summary)

A critical challenge of solid-state batteries is Li-filament penetration. Here, by quantifying microstructural properties and employing modeling techniques, the authors provide insight into solid-state battery failure modes and offer design guidelines to enhance safety and performance.

## Introduction

Among alternatives to conventional Li-ion batteries, solid-state batteries (SSBs) show potential for higher energy density and improved safety because they may enable the use of Li-metal anodes and replacement of flammable liquid electrolytes with solid electrolytes (SEs)^1,2^. Computational^3,4^ and experimental efforts have led to the discovery of SE materials including Li_7_La_3_Zr_2_O_12_ (LLZO)^5^, ${{{{{\rm{L}}}}}}{{{{{{\rm{i}}}}}}}_{2}{{{{{\rm{S}}}}}}-{{{{{{\rm{P}}}}}}}_{2}{{{{{{\rm{S}}}}}}}_{5}$ (LPS)^6^, and ${{{{{\rm{L}}}}}}{{{{{{\rm{i}}}}}}}_{6}{{{{{\rm{P}}}}}}{{{{{{\rm{S}}}}}}}_{5}{{{{{\rm{X}}}}}}$^7^ with high ionic conductivities comparable to those of liquid electrolytes (~10^−3^ S/cm). However, further improvement of the processability and scalability of these materials is critical for their commercialization^8^. The processing conditions of SEs, such as the fabrication pressure and processing temperature, significantly affect the measured ionic conductivity^9^, likely leading to the large discrepancies in reported values^10^. The ionic conductivity of LLZO increases from 10^−6^ to 10^−4^ S/cm upon increasing the sintering temperature from 1000 °C to 1150 °C, with a corresponding decrease in the porosity from 6.59% to 4.52%^11^. Likewise, the conductivity of LPS increases from 3 × 10^−4^ to 1.1 × 10^−3^ S/cm when increasing the fabrication pressure during hot pressing (200 °C) from 47 MPa^12^ to 270 MPa^13^, with a corresponding increase in the relative density from 85% to 98%.

Intuitively, optimizing the relative density should not only increase the ionic conductivity of the SE but also suppress Li-dendrite growth. However, extensive studies have demonstrated Li-filament penetration of LPS^14^ and LLZO^15^ regardless of their density or crystallinity^16,17^. Many studies have indicated that the penetration may be related to low ionic conductivity at grain boundaries^18^, inhomogeneous plating at the Li metal/SE interfaces^19^, electronic conductivity in the SE^20,21^, low relative density of the SE^22^, and pre-existing microstructural defects (such as cracks and pores) on the surface of and in bulk SEs^23^; however, consensus on the mechanism in various SEs has not yet been reached. As most of these factors are influenced by the fabrication conditions, it is important to quantify the effect of processing parameters such as the densification pressure on the micro- and macrostructural properties (e.g., porosity, tortuosity, pore networks) of the SE and on the failure of SSBs due to Li-filament growth.

In this work, we provide insight on the failure mechanism of SSBs due to Li-filament growth by investigating the effect of the fabrication pressure on the micro- and macrostructure of LPS. We first show that fully dense LPS SE (relative density >99%) is produced at fabrication pressures above 600 MPa. The ionic conductivity increases linearly during densification of the LPS pellet, as reported in similar studies^12,13^. However, the failure behavior of SSB cells as a function of densification is found to be more complicated: a symmetric cell (Li|LPS|Li) fails much faster as the density of the LPS pellet increases, before reaching a critical relative density (~95% for a fabrication pressure of 500 MPa) beyond which cell failure does not occur. To explain this highly non-linear failure behavior the micro- and macrostructure (pore size and connectivity, porosity, tortuosity) of LPS pellets are quantified using surface scanning electron microscopy (SEM), focused ion beam (FIB)–SEM tomography, and pellet-permeability tests, revealing that the pore networks formed during processing play a key role in the failure of SSBs. Our modeling results confirm the much higher Li-filament growth rate (and therefore faster cell failure) in denser pellets because of the much smaller pore sizes.

## Results

**Fig. 1:**
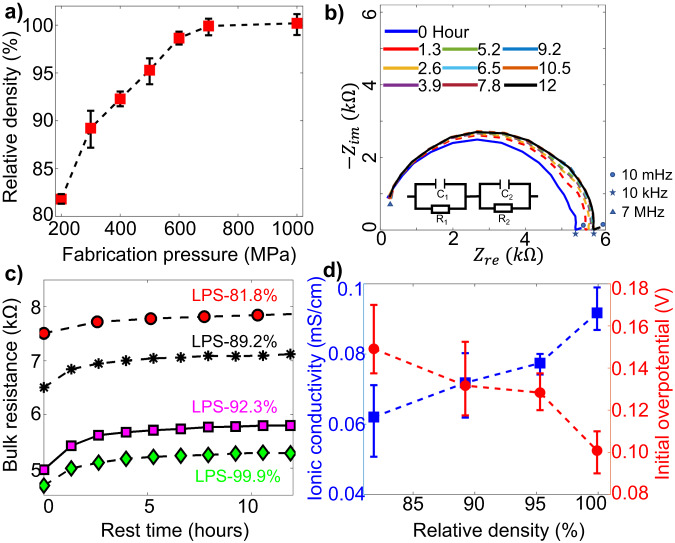
Effect of pellet density on cell resistance; error bars are defined as standard deviation. **a** Relative density of LPS pellet for various fabrication pressures (theoretical LPS density of 1.88 g/cm^3^). **b** Nyquist plots from sequential EIS measurements (with time interval of 1.3 h) of Li|LPS|Li symmetric cell (with LPS pellet relative density of ~89.5%). All the intermediate curves (dashed lines) are enveloped by the initial (*t* = 0 h, blue solid line) and final (*t* = 12 h, blue solid line) curves. **c** Temporal evolution of bulk (SE + SEI) resistance of symmetric cells with four different LPS relative densities. **d** Ionic conductivity (blue curve) of LPS pellets and the initial overpotential (red curve) of the cell at current density of 0.2 mA/cm^2^.

Figure 1a confirms that the relative density of the LPS pellet was well controlled by the fabrication pressure, with an almost fully dense LPS pellet (99.9%) obtained at a fabrication pressure of 700 MPa, consistent with previous results^13^. Figure 1b presents Nyquist plots from sequential electrochemical impedance spectroscopy (EIS) measurements of the LPS pellet with a density of 95.3% (LPS-95.3%). The real part of the impedance increases for the initial 2 h, before leveling off for the remaining 10 h, behavior consistent with a chemical reaction between LPS and Li metal^24^ and early passivation of the solid-electrolyte interphase (SEI)^15,25^. Figure 1c clearly shows the strong effect of the pellet density on the bulk (SE + SEI) resistance and its temporal evolution as well as passivation of all the cells during the initial 2 h. As depicted in Fig. 1d, the ionic conductivity linearly increases with increasing LPS density, and the initial overpotential decreases^12^. The increase in conductivity is likely related to the increase in contact area between the LPS particles at higher density.

**Fig. 2:**
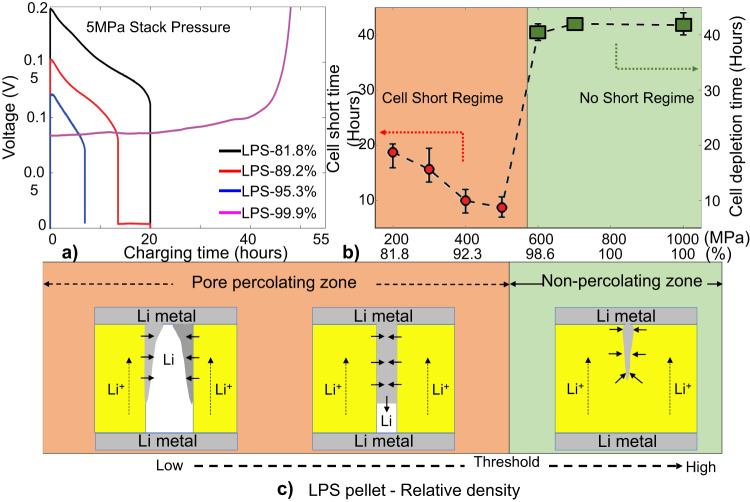
Effect of pellet density on Li-filament growth; error bars are defined as standard deviation. **a** Charging voltage of cells with different LPS pellet densities. **b** Cell-shorting time (“cell-shorting regime”) as a function of fabrication pressure and LPS relative density and threshold where the cell voltage increases rapidly (“no-short regime”). **c** Schematic of Li deposition within pores of LPS pellets with different pellet densities. Symmetric cells in the “percolating regime” have pore networks connecting two electrodes in the initial microstructure, whereas those in the “non-percolating regime” have no connecting pore network initially.

The effect of the LPS pellet density on Li-filament growth was investigated through electrochemical (EC) measurements. A total of 35 cells (5 cells per LPS density) were charged with a current density of 0.2 mA/cm^2^ until failure of the cell or depletion of the counter electrode. Failure occurred when the voltage dropped to zero and depletion occurred when the voltage rapidly increased to the cutoff value (1 V). Figure 2a presents the voltage curves of four representative cells with different LPS pellet densities. Cell shorting took longer for LPS-81.8% (18 h) than for LPS-89.2% (16 h) and LPS-95.3% (8 h), whereas depletion was observed in the LPS-99.9% cell after charging for >40 h.

Figure 2b summarizes the effect of the LPS pellet density on the cell survivability. Each data point is the average of 5 measurements, with the detailed EC results for each cell presented in Fig. SI-4. As the LPS relative density increases from 81.8% to 95.3%, the cell-shorting time decreases; however, cell shorting does not occur above a pellet density of 95.3%. Instead, depletion was observed in all the LPS 98–100% cells, as highlighted in Fig. SI-5, with a clean surface of the counter electrode (stripping side) at the end of the EC experiments.

Figure 2c presents a schematic of our hypothesis explaining the non-monotonic relation between the cell-shorting time and the LPS pellet density. At lower density, the LPS pellet is more porous with larger and interconnected pores, whereas the pores become smaller and more isolated at higher density^11^. As a result, a pore network connecting two electrodes may exist in low-density LPS pellets, indicated as the “pore-percolating zone” (orange area in Fig. 2c), whereas in high-density pellets, the pores become non-percolating (green area in Fig. 2c). These microstructural features are responsible for the shorting behavior of the symmetric cells observed in Fig. 2b: (1) Li filaments propagate easily in the percolating pores until shorting occurs when the LPS density is low (pore-percolating regime); however, propagation is suppressed by the non-percolating pores when the LPS density is in the non-percolating zone. (2) Within the pore-percolating regime, Li filaments propagate faster with decreasing pore size, causing faster cell shorting because of the reduction in the fillable volume.

**Fig. 3:**
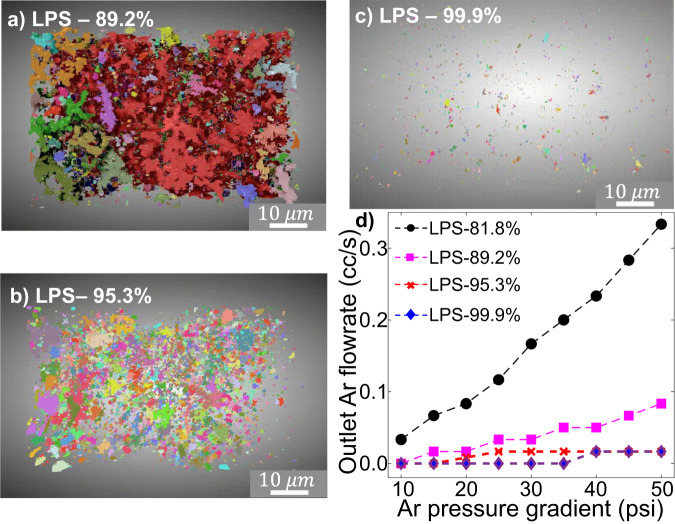
Characterizations of micro- and macrostructure of LPS. 3D structure of pores within the LPS pellets at densities of (**a**) 89.2%, (**b**) 95.3%, and (**c**) 99.9%. **d** Flowrate of Ar gas flowing out of LPS pellet as a function of Ar pressure gradient across the pellet.

To validate the aforementioned hypothesis, quantitative analyses of the pore microstructure and connectivity were performed using FIB–SEM tomography and permeability tests. The pore structures in the (50 μm)^3^ volume from different pellets (LPS-89.2%, LPS-95.3%, LPS-99.9%) are shown in Fig. 3a–c, respectively, with the different colors representing different interconnected pore networks. The pores are large and well connected in the LPS-89.2% cube, as exemplified by the largest pore network shown in red occupying most of the total pore volume. In contrast, the pores in the LPS-95.3% cube are small and connected, whereas the pores in the LPS-99.9% cube are small and isolated. The pore size, porosity, and connectivity (defined as the ratio between the largest pore volume and the total pore volume) of the three cubes were calculated statistically and are summarized in Table 1, with details provided in Figs. SI-6 and SI-7.

**Table 1:** FIB/SEM characterization analysis

Bulk LPS pellet density (%)	89.2	95.3	99.9
Porosity (%)	8.43	2.28	0.01
Connectivity (%)	76.8	4.88	3.34
Pore size (µm)	0.5–1.5	0.2–0.8	<0.1

Figure 3d depicts the relation between the outlet Ar gas flowrate Q and the pressure gradient $(\triangle P={P}_{0}-{P}_{{atm}})$ across LPS pellets with varying densities, showing that a higher Ar gas flux was measured for LPS-81.8% and LPS-89.2%, whereas little to no Ar gas flux was measured for LPS-95.3% and LPS-99.9%. These results confirm the presence of percolated pores in the low-density pellets and non-percolated pores in the high-density pellets. A more quantitative permeability analysis based on the modified Darcy’s law^26,27^ in the SI (The permeability tests and analysis) shows that their respective permeability values are ${k}_{82\%}=1.50\times {10}^{-3}{{{{{\rm{\mu }}}}}}{{{{{{\rm{m}}}}}}}^{2}$, ${k}_{89\%}=3.86\times {10}^{-4}{{{{{\rm{\mu }}}}}}{{{{{{\rm{m}}}}}}}^{2}$, ${k}_{95\%}=1.82\times {10}^{-5}{{{{{\rm{\mu }}}}}}{{{{{{\rm{m}}}}}}}^{2}$, and ${k}_{99\%}\approx 0$.

**Fig. 4:**
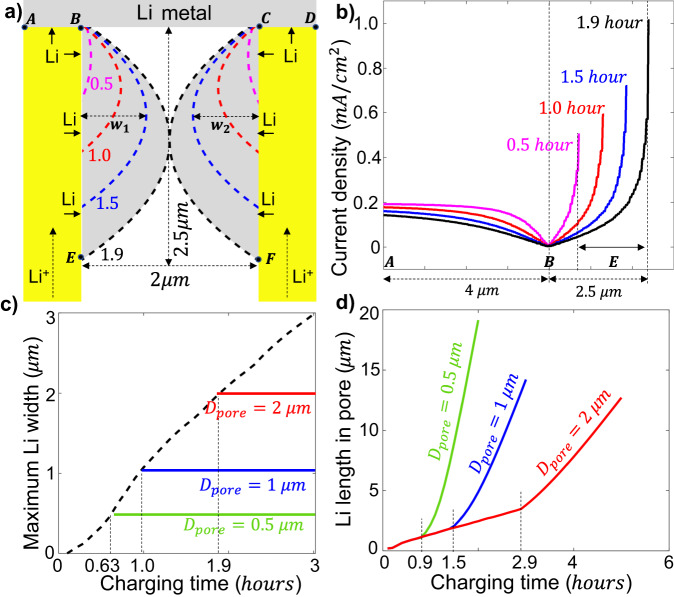
Simulation of Li deposition in SE pores. **a** Boundaries (dashed lines) of Li filament (gray area) at different charging times (in h) within a pore of 2-μm diameter. The Li metal on the top is the anode during charging. The maximum Li width at a specific time is the summation of the Li width on each side (*W* = *w*_1_ + *w*_2_). **b** Deposition current density along line A–B–E at different charging times. The labels “A”, “B”, and “E” along the x-axis correspond to locations A, B, and E in 4a. Note that location E changes at different time steps. **c** Maximum Li width in the pore when *D*_*pore*_ = 10 μm (dashed black line), *D*_*pore*_ = 2 μm (red line), *D*_*pore*_ = 1 μm (blue line), and *D*_*pore*_ = 0.5 μm (green line). **d** Length of Li filament in the pore when *D*_*pore*_ = 2 μm (red line), *D*_*pore*_ = 1 μm (blue line), and *D*_*pore*_ = 0.5 μm (green line).

Electro-chemo-mechanical modeling was employed was employed to quantify the Li propagation rate within pores in SE pellets^21,28^. Li-ions conduction in the SE is described by Ohm’s relation, the Li electrodeposition on SE/anode interface is described by the Butler-Volmer relation, and the Li deformation in the SE pore is described by visco-elastoplastic mechanics^29,30^. More details are provided in the Method section. Figure 4a presents a simplified model to describe the growth of Li (gray area) within a pore (white area) present in the LPS pellet (yellow area) near the Li-metal anode. Li ions are stripped from the counter electrode at the bottom, conducted through the SE, and deposited at the interface between the LPS and the top Li-metal electrode (line AB and CD) and the surface of the pore (line BE and CF). Li initially deposits at the three-phase corner at location B and C, where both Li^+^ ions (from LPS) and electrons (from the Li metal anode) are available for the reducing reaction (${{{{{{\rm{Li}}}}}}}^{+}+{{{{{{\rm{e}}}}}}}^{-}\to {{{{{\rm{Li}}}}}}$). The four dashed lines represent the Li boundary at four charging times (0.5, 1.0, 1.5, 1.9 h) under a current density of 0.2 mA/cm^2^. The metallic Li grows both radially and longitudinally toward the counter electrode. For example, it takes 1.9 h to close a 2-μm-diameter pore while growing to a depth of 2.5 μm in the LPS pellet. Figure 4b shows the deposition current along the interface (line A–B) and the pore surface (line B–E) at the four charging times, with the detailed distribution of ionic current and overpotential within the SE shown in Fig, SI-11. The current reaches a maximum at the advancing tip of the Li filament (location E) due to the lower resistance to reach this point. This maximum current density increases as the Li filament grows towards the counter electrode, from 0.5 mA/cm^2^ at ts0.5 h to 1.0 mA/cm^2^ at *t* = 1.9 h. It should be noted that this local current density is five times the nominal current.

When the Li width (*W* = *w*_1_ + *w*_2_) reaches the pore size (D_pore_) the radially deposited Li will start to extrude longitudinally. For example, when *t* > 1.9 h in Fig. 4a, the newly deposited Li extrudes longitudinally, which accelerates the Li-filament propagation. Figure 4c shows the starting time of Li extrusion for three different pore diameters: 0.5 μm (green line), 1 μm (blue line), and 2 μm (red line), which takes 0.63, 1.0, and 1.9 h, respectively. Figure 4d presents the propagation length of the Li filaments perpendicular to the electrodes within these pores. These results confirm the important fact that Li filaments indeed grow much faster towards the opposite electrode for smaller pore sizes. But the growth appears bilinear, rather than linear in time due to the changing mechanism as the pore is filled. Notably, the Li extrusion starts at *t* = 1.9 h in the 2-μm pore but accelerates at *t* = 2.9 h (Fig. 4d). This is because the extruded Li needs to first fill in the void space (such as the area in B–C–F–E in Fig. 4a), which takes 1 h for the 2-μm pore. The stable Li growth rate r_Li_ after the acceleration can be obtained from Fig. 4d, with the maximum (20.7 μm/h) observed for the smallest pore (0.5 μm), followed by 10.3 and 5.1 μm/h for the 1-μm and 2-μm pores, respectively. This Li growth rate from the simulation can be directly compared with the cell-shorting time presented in Fig. 2b.

## Discussion

Our experimental observations in Fig. 2b demonstrate the non-monotonous behavior of shorting time with pellet density. This finding implies that two different density regimes exist: In the percolating-pore regime a higher degree of densification actually leads to more rapid shorting as the volume of pores that needs to be filled decreases. Beyond a critical density where pores no longer percolate, simple growth of Li filaments through the conductor pellet is no longer possible and other mechanisms need to become active to shorten the cell. Indeed, while we find no shorting at low current density for the densest pellet, shorting of the cell can still be achieved at high current density, implying that in the non-percolating regime current-dependent mechanisms, such as stress build up^31^ or internal Li deposition due to electron leakage^21,32^ may contribute to failure. It should be noticed that the SE surfaces also vary with LPS relative density, as described in Fig. SI-6. This surface variation may affect the nucleation and the initial growth of Li filaments, but in our case this does not appear to modify the cell short-circuiting. This is likely since most Li filaments grow into existing pores in the bulk SE and this growth lasts until the cell failure.

**Fig. 5:**
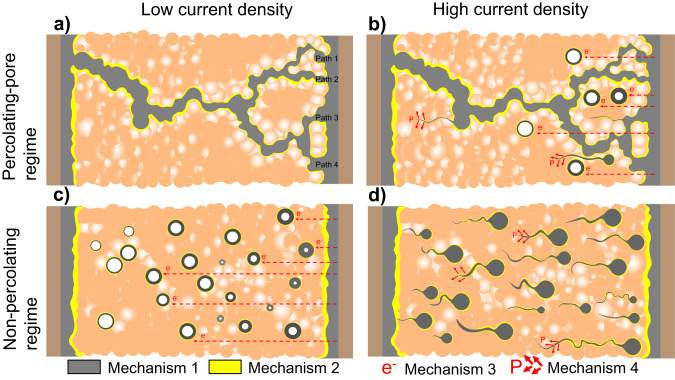
Schematics of Li-filament (Gray) growth in the solid electrolyte (Orange) at different conditions. **a** Li (gray) deposit within the tortuous pore network at low current density with multiple branches and varying thickness. The yellow line represents the SEI layer formed due to the chemical reaction between LPS and the Li filament. **b** Li (Gray) deposit within the tortuous pore network and isolated pores at high current density. **c** Li deposit in isolated pores at low current density. **d** Li deposits in isolated pores and causes fracture at high current density. Four identified mechanisms in the symmetric SSB: percolating pores (Mechanism 1), chemical reaction (Mechanism 2), electronic conductivity (Mechanism 3), and SE fracture (Mechanism 4). Li metal, the SEI layer, and voids are colored gray, yellow, and white, respectively. The dashed lines for Mechanism 3 represent electron conduction due to electronic conductivity of the SE. The red arrows for Mechanism 4 represent the fracture directions of the SE due to the development of hydrostatic pressure P.

Our modeling results, incorporating both transport and mechanics confirm the hypothesis built from the experiments: Pore walls are observed to fill with lithium in a droplet-like geometry grows counter to the Li+ flow, along a distance that is multiple times the pore diameter. For example, the 0.5 μm pore fills over a length of 20 μm even after only 1.5 h charging at 0.2 mA/cm^2^. It is challenging to make a quantitative comparison between our simulations and experiments as in reality percolating pore networks are tortuous, have varying radius along the path, and may join and branch, as shown schematically in Fig. 5 and for a real pellet in Fig. SI-10. However, the general conclusion drew from the model still hold larger pores in the SE enable the cell survives longer time than smaller pores.

Although this paper emphasizes the importance of pellet density on SSB failure due to Li-filament propagation through percolating pores, it should be noted that this is not the only mechanism but simply an easier path for Li-filament growth compared with SE fracture^33^, electrochemical reaction^34^, and SE electronic conductivity^32^. The four different mechanisms that have been identified as being responsible for Li-filament growth within the solid-state cell are illustrated in Fig. 5: (1) propagation through a percolating pore network, (2) growth of the SEI due to chemical reaction of SE and Li, (3) isolated Li deposition due to electronic conductivity of the SE, and (4) Li penetration and SE fracture at higher current density. Mechanism 1 (percolating pores) discussed in this paper is the most prevalent cause of SSB failure because most SE pellets used in the literature never reach the required threshold density (>95%), as discussed in Fig. 2. Lithium filaments prefer to propagate inside the percolating pores first because very low overpotential is needed for the growth. Mechanism 2 (chemical reaction) is present when the SE material is chemically unstable against Li metal, as is the case for all sulfides and some oxides. It has been reported that SSB failure can occur via this mechanism if an unstable SEI layer is formed^34^. Our results in Fig. 1c indicate that the chemical reaction between the Li metal and LPS pellet occurs in the first 2 h but stops after a stable SEI layer is formed. Therefore, this mechanism has limited effect in our study. Mechanism 3 (electronic conductivity) enables Li-metal deposition in the isolated pores in the SE, which eventually become interconnected and short the SSB once the percolating point is reached^21,32^. Given the non-negligible electronic conductivity (~10^−4^ mS/cm) of LPS we performed a control experiment using a buffer layer of Li_3_N + LiF^24^ to limit the possible transfer of electrons into the SE. The results shown Fig. SI-12 produced similar observations as those in Fig. 2 indicating that electron conductivity plays a minor role when the pellet is in the percolating pore regime. Mechanism 4 (SE fracture) can be observed when the SE material has low fracture toughness, and a high current density is applied^35^. As a large overpotential (or mechanical stress) is needed to trigger this mechanism, SE fracture is not likely to occur when a percolating pore network pre-exists in the LPS pellet but may become important for pellets in the “non-percolating regime” at escalated external current. Our control experiment in Fig. SI-13, confirms that a short circuit can still occur in dense pellets without percolating pores (LPS-99.2% and LPS-99.9%) under higher applied current densities (3.2 and 6.4 mA/cm^2^, respectively), further pointing at a current-dependent failure mechanism in this regime.

While these four mechanisms are usually entangled, it is worth noting that Mechanism 2 (chemical reaction), Mechanism 3 (electronic conductivity) and Mechanism 4 (SE fracture) are related to the material properties of the selected SE, whereas Mechanism 1 (percolating pores) is dependent on the microstructure of the SE pellet. Therefore, the detailed observations highlighted in the Results section are not exclusive to sulfide battery systems. The threshold relative density ( ~ 95% for the LPS used in this work) for closing the percolating pore network is a general requirement for the SE pellets made from other SE materials. This discovery explains the puzzling results reported in many works that Li dendrites still propagate into SEs even when the SE pellets are very dense. For example, it was reported that LLZO, even with a density close to that of a single crystal, can still have Li filaments propagating through^36^.

A straightforward solution to prevent percolating pores in SE pellets is to prepare a sufficiently dense pellet using various fabrication methods (cold pressing, hot pressing, high-temperature sintering, etc.). However, it can be challenging to densify certain SE materials, such as oxide materials. Unlike sulfides, which are soft enough for cold pressing, oxide SEs require high sintering temperature to achieve good densification. Several solutions for the densification of ceramics have been investigated with no breakthroughs yet, making the path to densification difficult^21,37^. Therefore, alternative methods should be explored. For example, the use of an interlayer or additives in the SE to reduce Li deposition in pores, or engineering on the pore connectivity in the SE to prevent the percolating network, etc.

The understanding of the mechanisms by which the current affects the propagation of Li within the SE is still puzzling. It is recognized that the growth of Li dendrites within the SE alters the distribution of local current, leading to an intensified current at the tip of the dendrite. Consequently, this results in a shift of the plating potential (the voltage at which plating, or deposition of Li occurs) inside the SE, deviating the 0 V potential^28,37–40^. However, the specific length scale at which this current focusing occurs in the lateral direction of the SE relative to the dendrite tip remains uncertain. Further investigation is necessary to determine the spatial extent over which the intensified current is concentrated around the dendrite tip.

A systematic investigation of the effect of LPS-pellet fabrication pressure (or relative density) on the failure mechanism of SSBs was performed. We showed that SSBs with denser SE fail more easily before a critical relative density is reached; after which failure is prevented. The most prevalent failure mechanism is the Li-filament growth in percolating pores within the SE, which is suppressed when the pores become isolated and small in high density pellets above the critical relative density (>95%). While different processing conditions (such as hot, warm, or cold pressing) may be required to obtain dense SE pellets, the critical relative density requirement for closing the percolating pore networks appears to be independent of the choice of SE material. Our study provides a quantitative guide for relative density optimization of SE pellet to prevent one of the most prevalent failure modes for Li-filament growth.

## Methods

### Preparation of Li|LPS|Li cell

Glassy LPS material was synthesized by ball milling 75% Li_2_ S (99.9% metal basis, Alfa Aesar) and 25% P_2_ S_5_ (99.9% metal basis, Alfa Aesar) for 4 h. The Li|LPS|Li cell was prepared in the following sequence. Symmetric lithium electrodes were prepared by rolling Li-metal chunks (99.9% metal basis, Alfa Aesar) onto copper foil. The cells were assembled using an in-house-designed cell-making toolkit (as illustrated in Fig. SI-1). A constant stack pressure of 5 MPa was applied to the cell to maintain the conformal Li–SE interfacial contact. Notably, the symmetric cell was assembled inside a small PEEK tube, which may be subjected to non-negligible deformation when very high pressing pressure is applied. This deformation changes the diameter of the cells accordingly; therefore, the final cell diameter should be calculated, with detailed simulation in Fig. SI-2.

### Electrochemical cycling and electrochemical impedance spectroscopy

Each cell was tested following two sequential stages: (1) Initial rest stage: the cells were rested for 12 h after assembly under the stack pressure. EIS measurement was conducted every 1.3 h to monitor the temporal evolution of both the bulk and interfacial resistances. (2) Electrochemical (EC) cycle stage. The charge, cycling, and EIS measurements were performed using a potentiostat (VMP-300, BioLogic). The EC and EIS measurements were conducted in a temperature chamber to ensure a constant cycling temperature. The EC measurements were performed using a current *i* = 15.84 μA, with an electrode diameter *d* = 3.175 mm to maintain the current density at $J=0.2\frac{{{{{{\rm{mA}}}}}}}{{{{{{\rm{c}}}}}}{{{{{{\rm{m}}}}}}}^{2}}$. The current was increased later in the investigation to adjust the current density criteria. The EIS measurements were conducted at a frequency ranging from 10^−3^ to 7 × 10^6^ Hz.

### Focused ion beam and scanning electron microscopy

FIB–SEM characterization was performed on an FEI Helios G4 dual-beam FIB system equipped with a Ga^+^ ion beam, as shown in Fig. SI-6. Consecutive slice milling and image acquisition were performed using the FEI Slice and View software. LPS pellets of varying densities were cut in the normal direction using FIB and characterized using SEM under a tilt angle of 52°.

Image processing and reconstruction: The resulting SEM image stacks were first rescaled to compensate for the 52° angle between the electron beam and sample cross-section. Then, several representative slices were selected, and manual segmentation of different components (LPS and void) was performed. The manually segmented images were used to train a classifier using the Trainable Weka Segmentation plug-in in the ImageJ software, which was then used to segment the entire image stack. All the 3D reconstruction and visualization were reconstructed using the Dragonfly software from 100 cross-sectional slides with each slice 50 nm thick.

### Permeability tests

The permeability tests were performed by pumping Ar gas through a custom-made piping and hose system, as shown in Fig. SI-9. The inlet flow of Ar gas was measured and compared with the outlet Ar gas flow, which was used to determine the permeability of the tested sample. The LPS pellet was sealed inside two cylindrical tubes, with Ar gas flowing into the pellet from the bottom tube and flowing out to the top tube. The pneumatic pressure (P_in_) of the inlet Ar gas in the bottom tube was controlled by the valve on the Ar tank. The flow rate (Q) of the outlet Ar gas in the top tube was measured using a highly sensitive flow meter (sensitivity of 0.01 cc/s). The pneumatic pressure (*P*_*out*_) of the outlet Ar was measured very close to the atmospheric pressure (*P*_*out*_ = *P*_*atm*_).

### Modeling of Li deposition in pores

Both the Li electrodeposition and interfacial contact loss are affected by the charge-transfer reactions (described by the Butler-Volmer relation), mass transfer in the SE (described by the Ohmic relation), and interfacial contact mechanics (described by elastoplastic continuum mechanics). A comprehensive approach combining Li electrodeposition, mass transport with elasticity and plasticity of both the Li metal and SE used in the model through coupled PDEs. A brief list of relevant PDEs is provided in the following, with a more detailed description of the individual physics and corresponding PDE in our earlier similar modeling work^21,28^.

Quasi-static mechanical equilibrium is assumed for both Li metal and the SE:

1 $$\nabla \cdot {{{{{\mathbf{\sigma }}}}}}=0$$

Linear elasticity is assumed for the elastic state of both Li metal and the SE:

2 $${{{{{\mathbf{\sigma }}}}}}{{{{{\boldsymbol{=}}}}}}\frac{E}{1+\nu }{{{{{\boldsymbol{\varepsilon }}}}}}{{{{{\boldsymbol{+}}}}}}\frac{\nu E}{\left(1+\nu \right)\left(1-2\nu \right)}{{{{{\rm{trace}}}}}}\left({{{{{\boldsymbol{\varepsilon }}}}}}\right){{{{{\bf{I}}}}}}$$

An elastic/perfect plastic model without hardening is assumed for the Li metal plastic flow, with the Von Mises criterion and associated flow rule:

3 $$\Phi \left({{{{{\mathbf{\sigma }}}}}}\right){{{{{\boldsymbol{\equiv }}}}}}\sqrt{\frac{3}{2}}\left|{{{{{\rm{dev}}}}}}\left({{{{{\mathbf{\sigma }}}}}}\right)\right|{{{{{\boldsymbol{-}}}}}}{\sigma }_{y}=0,{{{{{\rm{d}}}}}}{{{{{{\boldsymbol{\varepsilon }}}}}}}^{{{{{{\rm{p}}}}}}}{{{{{\boldsymbol{=}}}}}}{{{{{\rm{d}}}}}}\lambda \frac{\partial \varPhi }{\partial {{{{{\mathbf{\sigma }}}}}}}$$

Since the SE is a single-ion conductor, the conduction is therefore purely ohmic:

4 $${\nabla }^{2}{\phi }_{{SE}}=0,{{{{{\bf{i}}}}}}=-{\sigma }_{{{Li}}^{+}}\nabla {\phi }_{{SE}}$$

At SE/Li metal interface, Butler-Volmer relation^35,41^ is employed as the boundary condition:

5 $${i}_{{ct}}={i}_{{exc}}{e}^{\frac{(1-{\alpha }_{a}){\bar{V}}_{{Li}}\triangle {P}_{{Li}}}{{RT}}}\left({{{{{{\rm{e}}}}}}}^{\frac{{\alpha }_{a}F}{{RT}}\eta }-{{{{{{\rm{e}}}}}}}^{-\frac{{\alpha }_{c}F}{{RT}}\eta }\right)$$

6 $${i}_{{ct}}=-{i}_{n}=-{{{{{\bf{i}}}}}} \cdot {{{{{{\bf{n}}}}}}}_{{{{{{\rm{SE}}}}}}}$$

An in-house-developed code based on the finite element method and the MOOSE framework^42^ was implemented to solve all the coupled electro-chemo-mechanical PDEs numerically. The default values of the parameters (such as electronic/ionic conductivities for Li^+^ transport in the SE and electrons in the Li metal) used in this work were obtained from experimental measurements of LPS-type SE and are listed in the last column of Table 2.

**Table 2:** Key parameters used in this work

Name	Symbol	Unit	Value	ref
Exchange current density at electrode/SE interface	${i}_{{exc}}^{k}$	mA/cm^2^	1.3	^43^
Exchange current density at void/SE interface	${i}_{{exc}}^{V}$	mA/cm^2^	0.01	^44^
Ionic conductivity in the SE	${\sigma }_{{M}^{+}}$	mS/cm	0.1	^45^
Electronic conductivity in the SE	${\sigma }_{{e}^{-}}$	mS/cm	10^−4^	^32^
Electric conductivity in the M metal	*σ*_*M*_	mS/cm	10^5^	
Fracture toughness of the SE	*K*_*c*_	${{{{{\rm{MPa}}}}}}\bullet \sqrt{{{{{{\rm{m}}}}}}}$	0.2	^46^
Bulk modulus of the M metal	*K*	GPa	11	^47^

### Supplementary information

Supplementary Information

Peer Review File

## Data Availability

The Electrochemical charging data generated in this study have been deposited in the Figshare database under accession code 10.6084/m9.figshare.24717789.

## Code availability

The code used for the simulation part in this study has been deposited in the Figshare database under accession code 10.6084/m9.figshare.24717789.

## References

[1] Janek J, Zeier WG (2016). A solid future for battery development. Nat. Energy.

[2] Famprikis, T., Canepa, P., Dawson, J. A., Islam, M. S. & Masquelier, C. Fundamentals of inorganic solid-state electrolytes for batteries. *Nat. Mater.* **18**, 1278–1291 (2019). 10.1038/s41563-019-0431-3 31427742

[3] Wang Y (2015). Design principles for solid-state lithium superionic conductors. Nat. Mater..

[4] Xiao Y (2021). Lithium oxide superionic conductors inspired by garnet and NASICON structures. Adv. Energy Mater..

[5] Taylor NJ (2018). Demonstration of high current densities and extended cycling in the garnet Li7La3Zr2O12 solid electrolyte. J. Power Sources.

[6] Han F, Yue J, Zhu X, Wang C (2018). Suppressing Li dendrite formation in Li2S‐P2S5 solid electrolyte by LiI incorporation. Adv. Energy Mater..

[7] Zhou L, Assoud A, Zhang Q, Wu X, Nazar LF (2019). New family of argyrodite thioantimonate lithium superionic conductors. J. Am. Chem. Soc..

[8] Wang MJ, Kazyak E, Dasgupta NP, Sakamoto J (2021). Transitioning solid-state batteries from lab to market: linking electro-chemo-mechanics with practical considerations. Joule.

[9] Lee J, Lee T, Char K, Kim KJ, Choi JW (2021). Issues and advances in scaling up sulfide-based all-solid-state batteries. Acc. Chem. Res..

[10] Randau S (2020). Benchmarking the performance of all-solid-state lithium batteries. Nat. Energy.

[11] Shen F, Dixit MB, Xiao X, Hatzell KB (2018). Effect of pore connectivity on Li dendrite propagation within LLZO electrolytes observed with synchrotron X-ray tomography. ACS Energy Lett..

[12] Garcia-Mendez R, Mizuno F, Zhang R, Arthur TS, Sakamoto J (2017). Effect of processing conditions of 75Li2S-25P2S5 solid electrolyte on its DC electrochemical behavior. Electrochim. Acta.

[13] Garcia‐Mendez R, Smith JG, Neuefeind JC, Siegel DJ, Sakamoto J (2020). Correlating macro and atomic structure with elastic properties and ionic transport of glassy Li2S‐P2S5 (LPS) solid electrolyte for solid‐state Li metal batteries. Adv. Energy Mater..

[14] Nagao M (2013). In situ SEM study of a lithium deposition and dissolution mechanism in a bulk-type solid-state cell with a Li 2 S–P 2 S 5 solid electrolyte. Phys. Chem. Chem. Phys..

[15] Ren Y, Shen Y, Lin Y, Nan C-W (2015). Direct observation of lithium dendrites inside garnet-type lithium-ion solid electrolyte. Electrochem. Commun..

[16] Swamy T (2018). Lithium metal penetration induced by electrodeposition through solid electrolytes: example in single-crystal Li6La3ZrTaO12 garnet. J. Electrochem. Soc..

[17] Zhang LC (2020). Intragranular growth and evenly distribution mechanism of Li metal in Li7La3Zr2O12 electrolyte. J. Power Sources.

[18] Sudo R (2014). Interface behavior between garnet-type lithium-conducting solid electrolyte and lithium metal. Solid State Ion..

[19] Sharafi A (2017). Surface chemistry mechanism of ultra-low interfacial resistance in the solid-state electrolyte Li7La3Zr2O12. Chem. Mater..

[20] De Jonghe LC, Feldman L, Beuchele A (1981). Slow degradation and electron conduction in sodium/beta-aluminas. J. Mater. Sci..

[21] Tu, Q., Shi, T., Chakravarthy, S. & Ceder, G. Understanding metal propagation in solid electrolytes due to mixed ionic-electronic conduction. *Matter*10.1016/j.matt.2021.08.004 (2021).

[22] Tsai C-L (2016). Li7La3Zr2O12 interface modification for Li dendrite prevention. ACS Appl. Mater. interfaces.

[23] Porz, L. et al. Mechanism of lithium metal penetration through inorganic solid electrolytes. *Adv. Energy Mater*. **7**, 1701003 (2017).

[24] Ji X (2020). Solid‐state electrolyte design for lithium dendrite suppression. Adv. Mater..

[25] Wenzel S (2016). Interphase formation and degradation of charge transfer kinetics between a lithium metal anode and highly crystalline Li7P3S11 solid electrolyte. Solid State Ion..

[26] Shugard, A. D. & Robinson, D. B. *A Simple Model of Gas Flow in a Porous Powder Compact* (Sandia National Lab, 2014).

[27] Wu Y-S, Pruess K (1998). Gas flow in porous media with Klinkenberg effects. Transp. porous Media.

[28] Tu Q, Barroso-Luque L, Shi T, Ceder G (2020). Electrodeposition and mechanical stability at lithium-solid electrolyte interface during plating in solid-state batteries. Cell Reports Physical. Science.

[29] LePage WS (2019). Lithium mechanics: Roles of strain rate and temperature and implications for lithium metal batteries. J. Electrochem. Soc..

[30] Chen Y (2020). Li metal deposition and stripping in a solid-state battery via Coble creep. Nature.

[31] Cheng E, Sharafi A, Sakamoto J (2017). Intergranular Li metal propagation through polycrystalline Li6.25Al0.25La3Zr2O12 ceramic electrolyte. Electrochim. Acta.

[32] Han F (2019). High electronic conductivity as the origin of lithium dendrite formation within solid electrolytes. Nat. Energy.

[33] Ning Z (2021). Visualizing plating-induced cracking in lithium-anode solid-electrolyte cells. Nat. Mater..

[34] Lee C (2021). Stack pressure measurements to probe the evolution of the lithium–solid-state electrolyte interface. ACS Energy Lett..

[35] Monroe C, Newman J (2005). The impact of elastic deformation on deposition kinetics at lithium/polymer interfaces. J. Electrochem. Soc..

[36] Golozar M (2020). Direct observation of lithium metal dendrites with ceramic solid electrolyte. Sci. Rep..

[37] Barroso-Luque L, Tu Q, Ceder G (2020). An analysis of solid-state electrodeposition-induced metal plastic flow and predictions of stress states in solid ionic conductor defects. J. Electrochem. Soc..

[38] Doux, J. -M., et al. Pressure effects on sulfide electrolytes for all solid-state batteries. *J. Mater. Chem.**A*. **8**, 5049 (2020).

[39] Hänsel C, Kundu D (2021). The stack pressure dilemma in sulfide electrolyte based Li metal solid‐state batteries: a case study with Li6PS5Cl solid electrolyte. Adv. Mater. Interfaces.

[40] Shi T (2020). High active material loading in all‐solid‐state battery electrode via particle size optimization. Adv. Energy Mater..

[41] Monroe C, Newman J (2004). The effect of interfacial deformation on electrodeposition kinetics. J. Electrochem. Soc..

[42] Permann CJ (2020). MOOSE: Enabling massively parallel multiphysics simulation. SoftwareX.

[43] Chiku M, Tsujiwaki W, Higuchi E, Inoue H (2012). Microelectrode studies on kinetics of charge transfer at an interface of Li metal and Li2S-P2S5 solid electrolytes. Electrochemistry.

[44] Tian H-K, Liu Z, Ji Y, Chen L-Q, Qi Y (2019). Interfacial electronic properties dictate Li dendrite growth in solid electrolytes. Chem. Mater..

[45] Bachman JC (2015). Inorganic solid-state electrolytes for lithium batteries: mechanisms and properties governing ion conduction. Chem. Rev..

[46] McGrogan FP (2017). Compliant yet brittle mechanical behavior of Li2S–P2S5 lithium‐ion‐conducting solid electrolyte. Adv. Energy Mater..

[47] Masias, A., Felten, N., Garcia-Mendez, R., Wolfenstine, J., & Sakamoto, J. Elastic, plastic, and creep mechanical properties of lithium metal. *J. Mater*. Sci. **54**, 2585–2600 (2019).

