# Supplementary material for: Effect of solid-electrolyte pellet density on failure of solid-state batteries
Source: Nat Commun. 2024 Jan 29;15:858. doi: 10.1038/s41467-024-45030-7 (PMC10825224; doi:10.1038/s41467-024-45030-7)
Supplement: Supplementary file 1 — Supplementary Information [file 41467_2024_45030_MOESM1_ESM.pdf]

Support Information

Effect of Solid-Electrolyte Pellet Density on Failure of Solid-State Batteries

Mouhamad S. Diallo[1†], Tan Shi[1], Yaqian Zhang [1], Xinxing Peng [1], Imtiaz Shozib[2], Yan Wang[3], Lincoln J. Miara[3], Mary C. Scott[1,4], Qingsong Howard Tu[2,5,†,*], Gerbrand Ceder[1,5,*]

1. Department of Materials Science and Engineering, University of California, Berkeley, CA 94720, USA

2. Department of Mechanical Engineering, Rochester Institute of Technology, Rochester, New York 14623, United States

3. Advanced Materials Lab, Samsung Advanced Institute of Technology-America, Samsung Semiconductor Inc., Cambridge, MA 02138, USA

4. National Center for Electron Microscopy, Molecular Foundry, Lawrence Berkeley National Laboratory, 1 Cyclotron Road, Berkeley, California 94720, USA

5. Materials Sciences Division, Lawrence Berkeley National Laboratory, Berkeley, CA 94720, USA

[†]The authors contributed equally

[*]These authors jointly supervised this work: Pr. Gerbrand Ceder (Email: gceder@berkeley.edu), Pr. Qingsong Howard Tu (Email: howard.tu@rit.edu)

Lead contact: Prof. Gerbrand Ceder (Email: gceder@berkeley.edu)

- **The experimental setup and the house-designed cell making toolkit**

Cells are assembled in a small PEEK tube (Figure SI-1a-b). SE (LPS) is first pressed in a PEEK small tube at different pressure, then Lithium metal is pressed at both side of the SE. Copper foils are then placed on both end of the Li-metal electrodes and then, stainless steel rods are used to press the cell. The cell is then placed in a testing setup (Figure SI-1c) which has a spring to control the stack pressure. the testing setup is then encapsulated in a sealed container to ensure the cell is running in an inert environment.

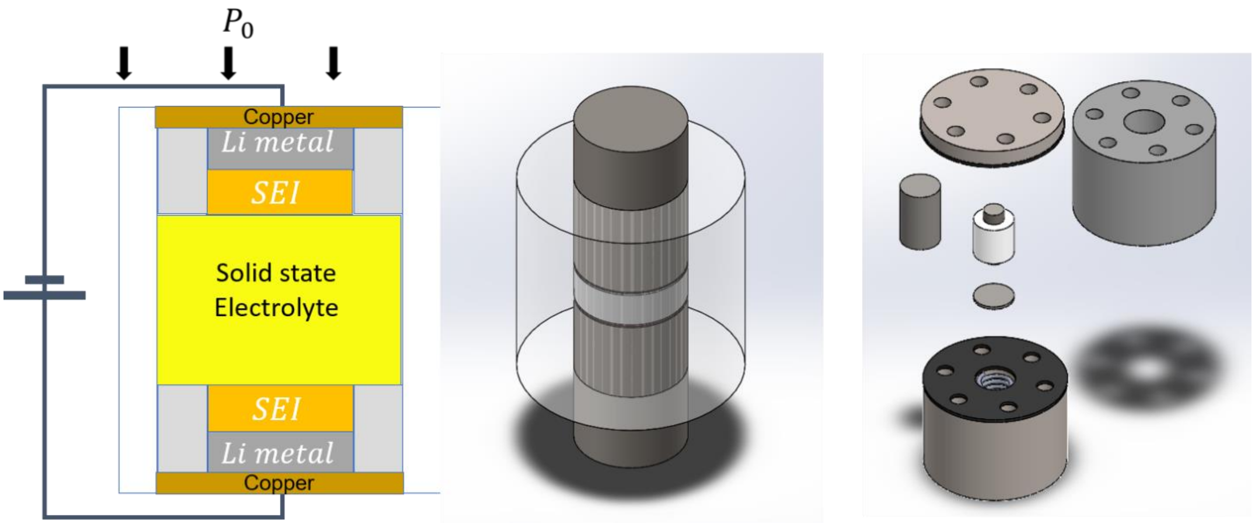

Figure SI-1. The experimental setup and the house-designed cell-making toolkit.

- **The deformation of cell diameter due to fabrication pressure**

The material for the die to make the LPS pellet is PEEK, which is subjected to non-negligible plastic deformation (6%–8%) under high uniaxial pressures; therefore, the final cell diameter (~6.8 mm), instead of the initial value (6.35 mm), is used for the calculation of density and conductivity (Figure 1d), with detailed calculation in *Figure SI-2*.

. This was then used to calculate the relative density of high-pressure cells.

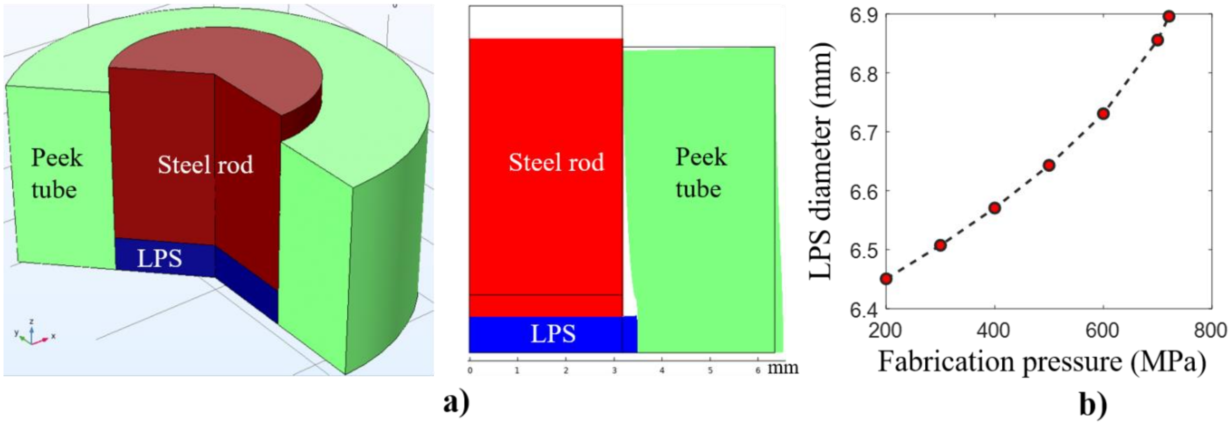

Figure SI-2. The change of LPS diameter due to deformation of the toolkit under high fabrication pressure. (a) The shape of each parts under fabrication pressure 700MPa; (b) the LPS diameters as a function of the fabrication pressure.

- **The optimization of stack pressure**

Stack pressure was applied on the cells as shown in *Figure-SI-1*. The cells were prepared with two different pressing pressure to evaluate the effect of stack pressure on both percolated and non-percolated pores pellet; LPS-89% has a percolated pore network and LPS-95% has isolated and non-percolated pores. The EIS results are plotted in *Figure SI-3*. We plot bulk resistance, extracted from the EIS data, vs resting time, which is the time is left without charging. We observe that for a stack pressure of 8MPa, the bulk resistance of the cells, in both the percolated and non-percolated range, decreases over time. This is because lithium metal is creeping in the LPS pellet via chemo-mechanical effects. The stack pressure applied on the SSB cell is a key parameter that affects the interfacial contact between the Li metal and LPS pellet. The interfacial resistance can be high if too low a stack pressure leads to contact loss [28], whereas SE fracture or Li extrusion into the SE pores can occur if the stack pressure is too high: [29] both cases are investigated and presented in *Figure SI-3* in which we show that our current experimental setup, 3-MPa stack pressure induces contact loss, whereas 8 MPa, too large, does not yield a stable interface. The optimal stack pressure in this work (~5 MPa) is different from that in related work in the literature *[28, 30]*, which may be due to the different wall frictions in different setups.

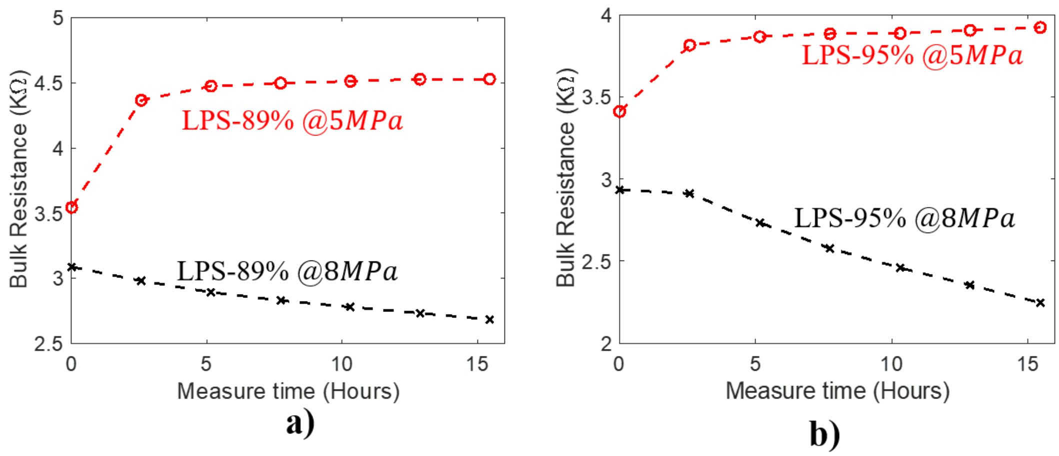

Figure SI-3. Stack pressure effect. a) Comparison of bulk resistance of cell with percolated pores (a, LPS-89%) and non-percolated pores (b, LPS-95%) under two stack pressure.

- **The EC curves of individual LPS pellet density**

The electrochemical dataset is plotted in *Figure SI-4* it shows all 35 cells measured with increasing density. The trend is summarized in the main body of the paper, in *Figure 2*. There are some noises present in the data, which is due to the sensitivity of the measurement devices, but it does not affect the results gathered.

It is worth noting that, for the high-density cells, which do not short, we show in Figure SI-4-e)-f) and -g) that, with varying amounts of Li-metal on the electrode the depletion time changes. We show that the depletion time can vary between 20 hours to beyond 140hours on a 600 MPa pressed pellet.

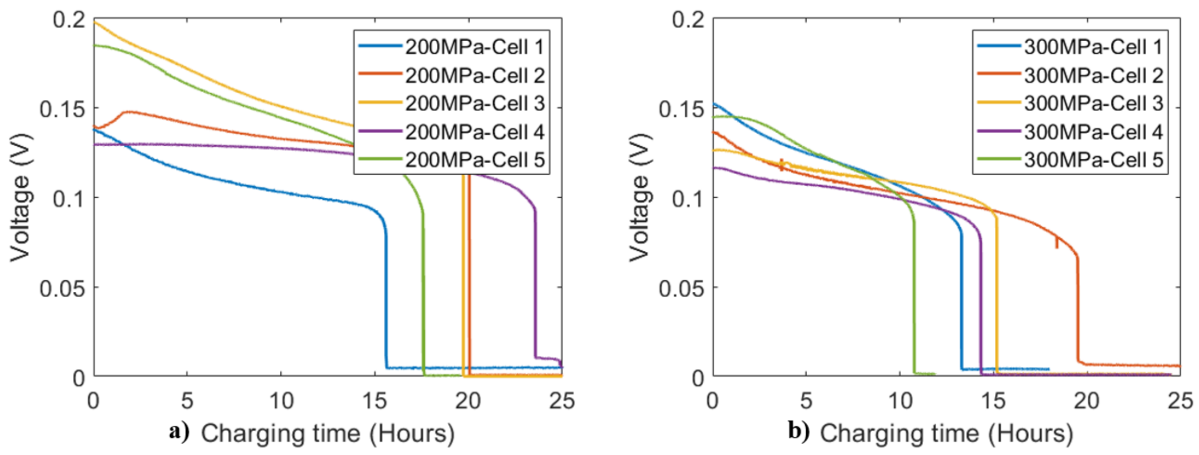

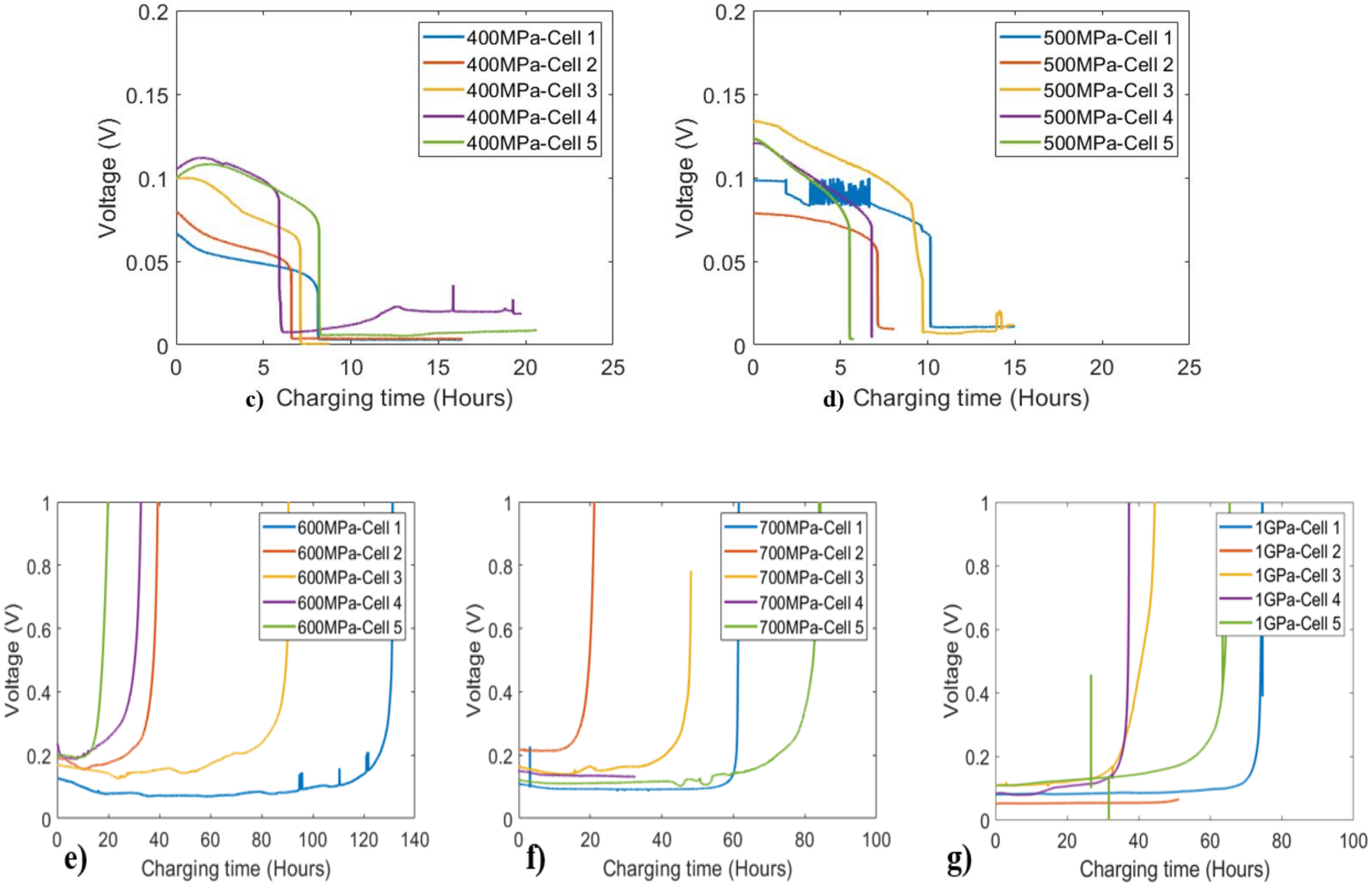

Figure SI-4. Electrochemical results of all cells at different fabrication pressure. a) at pressure 200MPa. b) at pressure 300MPa. c) at pressure 400MPa. d) at pressure 500MPa. e) at pressure 600MPa. f) at pressure 700MPa. g) at pressure 1000MPa

- **The clean surface at counter electrode showing the depletion**

The cells which have high density, greater than 95%, are determined to be non-percolating, which means the LPS pellet has pores that small and not connected. It is shown that, based on the lithium amount in the stripping side, the cells can survive more than 140 hours charging until depletion of the lithium. *Figure SI-5* shows two disassembled LPS pellets, one pressed at 700 MPa and the other at 1 GPa. For both cells both the lithium stripping side and plating side are presented, and lithium metal is fully depleted from the stripping side.

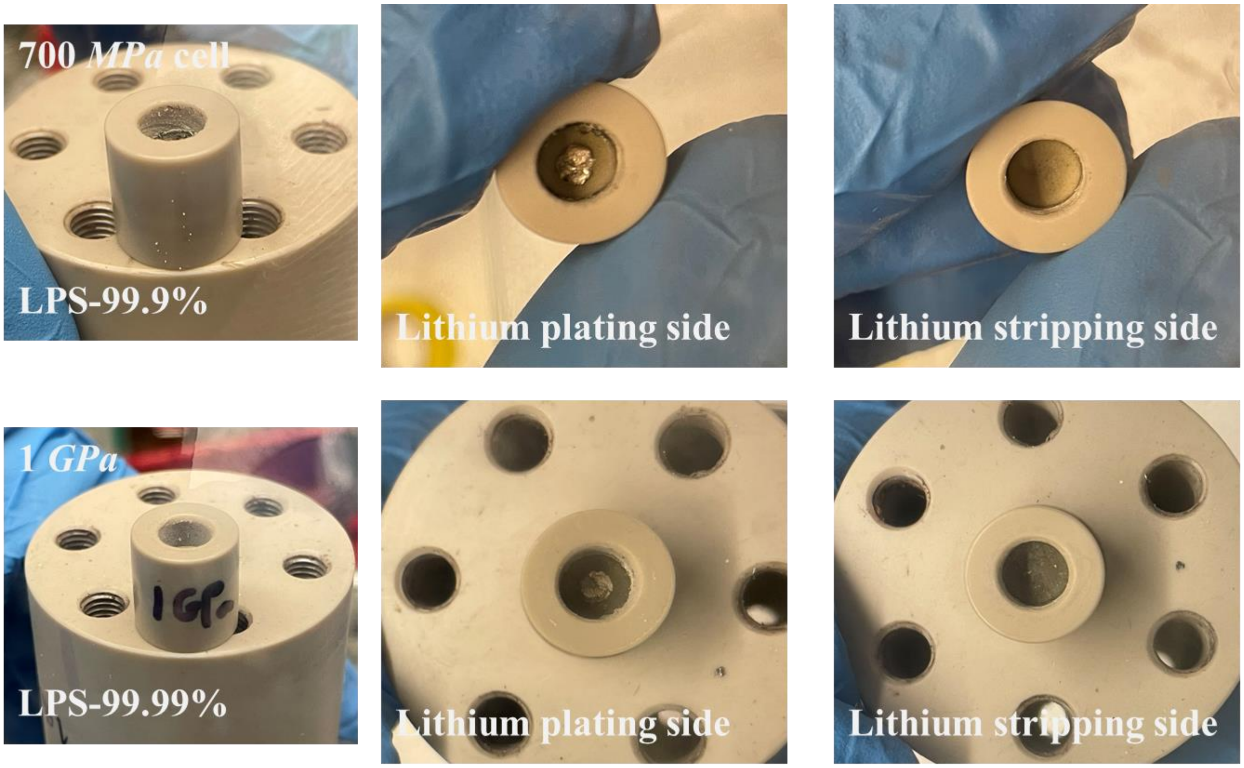

Figure SI-5. The LPS surfaces at the plating side and at the stripping side of the 700MPa and 1GPa cells. The shiny metal at the plating surface is the 1/8-inch lithium metal.

- **High-resolution tomography reveals local microstructure in the bulk LPS pellet**

LPS pellets of varying densities were cut in the normal direction using FIB and characterized using SEM under a tilt angle of 52°.The 3D pore microstructure at the bottom right (highlighted in yellow) was reconstructed from 100 cross-sectional slides along the milling direction. Figure SI-6 describes the general procedure of the characterization. The LPS pellet was cut in the normal direction with FIB and characterized with SEM tilted by

an angle θ (top-left image). The secondary electron image (top-right image) of the cross section (with size $50\mu m \times 50\mu m$) was captured and analyzed with the image processing software Dragonfly. [37] Multiple cross-sectional slides along the "milling direction" were obtained and analyzed following the same procedure, with final images shown in the bottom-left. The black area in the sequential images represent the pores and the gray area is the LPS material. The 3D structure is constructed from these images (bottom-right image). Pores are highlighted in yellow and LPS material in blue.

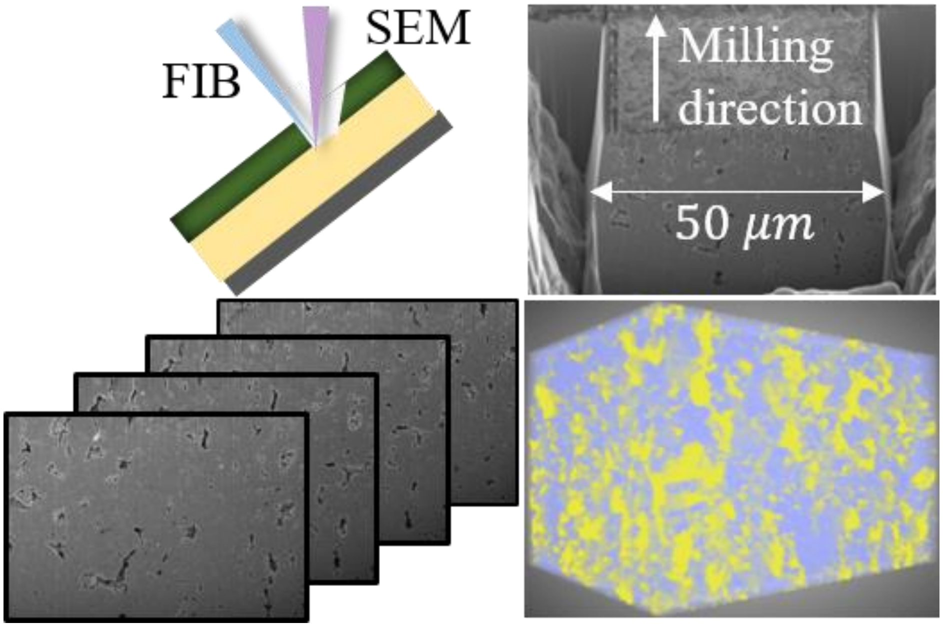

Figure SI-6 General characterization procedure

A house-developed MATLAB code is applied to the 3D tomography images for a more quantitative analysis. The pore size, porosity, and the connectivity (defined as the ratio between the largest pore volume over the total pore volume) of the three pellets in Figure 3 are calculated statistically in *Figure SI-7*.

In summary, a range of pore size with $1\sim2\ \mu m$, $0.2\sim0.8\ \mu m$ and $< 0.1\ \mu m$ are observed in the LPS-81.3%, LPS-89.5%, and LPS-100%, respectively. The porosity of these three pellets

are 8.43%, 2.28%, and 0.02%, respectively; and their connectivity values are 76.8%, 4.88%, and 3.34%, respectively.

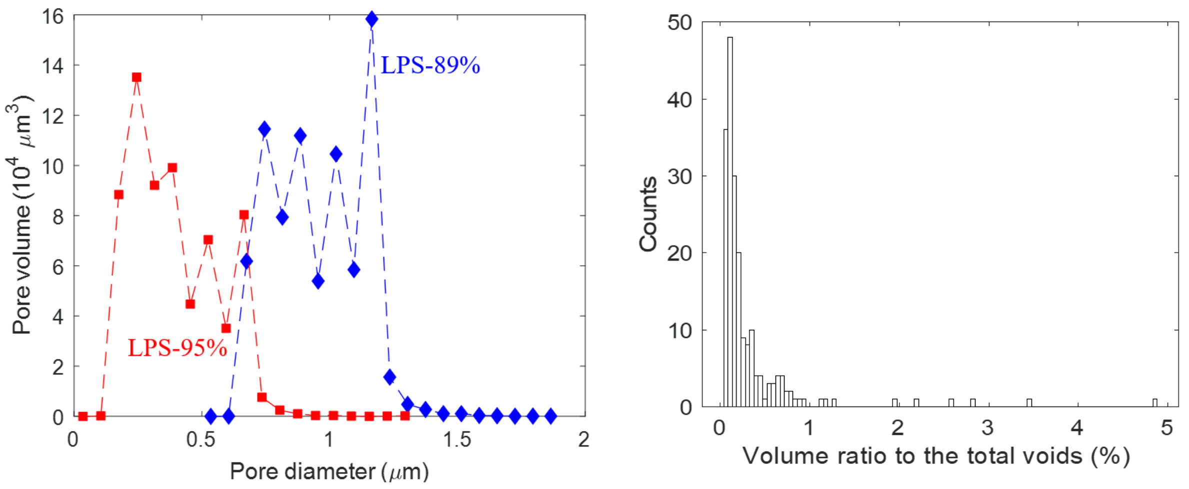

a) b)

Figure SI-7. The statistics of voids in pores with different densities.

- **SEM on the LPS pellet surface**

SEM were used on LPS pellets with different densities to characterize surface morphology. *Figure SI-6* shows SEM images of the surface of LPS pellets at three different relative densities (LPS-89.2%, LPS-95.3%, and LPS-99.9%). It clearly shows that the pore size on the pellet surface decreases as the pellet becomes denser. Pores with diameter larger than 2 μm are frequently observed on the surface of LPS-89.2% pellet. These pores are not only

interconnected on the surface, but also deep into the pellet (characterized by the dark area within the pores). In the LPS-95.3% pellet, both the size ($1 \sim 2\ \mu m$) and the number of connecting pores are reduced. Some pores are deep into the pellet while some only exist on the surface layer (characterized by the gray area within the pores). No connected pores (with diameter $< 1\ \mu m$) were found on the surface of the LPS-99.9% pellet, and all these pores are shallowly presented on the surface layer of the pellet. Notably, the images obtained can only represent the microstructure on the surface (or a thin surface layer) of the pellet. While the bulk microstructural properties inside the pellet may follow similar trend as that on the surface, more quantitative measurements are still needed.

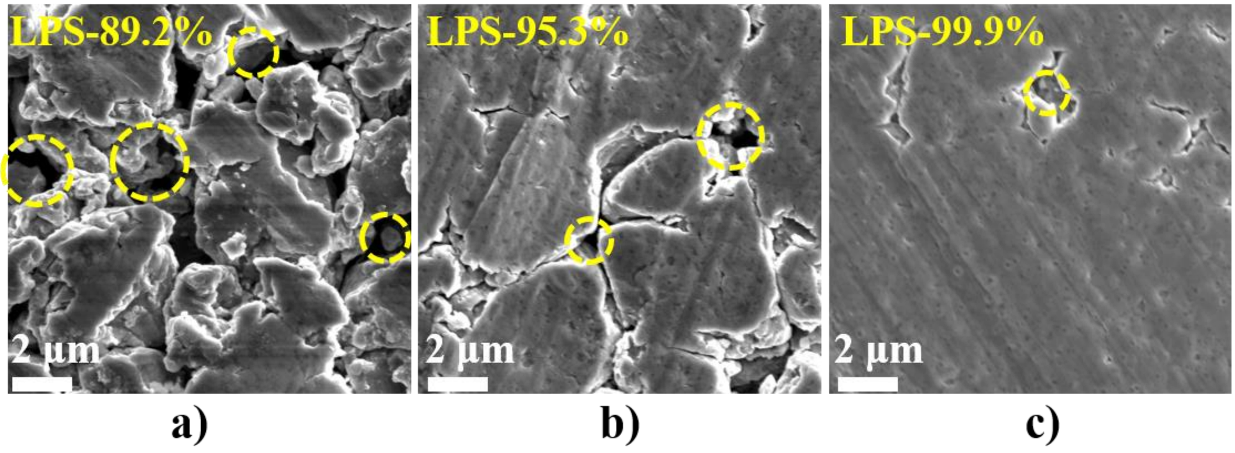

Figure SI-8. The SEM results on the surface of LPS pellets.

- **The permeability tests and analysis**

The pore connectivity was also quantified using the apparatus shown in Figure SI-8.

The quantitative pellet permeability can be calculated using the modified Darcy's law by considering the Klinkenberg effect due to the compressible Ar [32, 33]. In summary, Darcy's equation describes the linear relation between the flowrate (Q) and the pressure drop (ΔP) across the pellet thickness ($L = 2mm$): $\frac{Q}{A} = -\frac{k}{\mu L}\Delta P$, where A is the cross-sectional area of the top tube,

k is the pellet permeability, and μ is the dynamic viscosity of the Ar gas within the LPS pellet ($\mu_{Ar} = 2.23 \times 10^{-5}\ Pa\ \cdot s$). [34] The permeability of the four pellets in Figure 3f can therefore be extrapolated as $k_{82\%} = 1.50 \times 10^{-3} \mu m^2$, $k_{89\%} = 3.86 \times 10^{-4} \mu m^2$, $k_{95\%} = 1.82 \times 10^{-5} \mu m^2$, and $k_{99\%} \approx 0$.

The pellet permeability (k) can further be related to the microstructural parameters through the Carman–Kozeny relation: $k = \frac{d^2}{72}\frac{(1-\rho)^3}{\rho^2\tau^2}$, where d is the pore diameter, ρ is the pellet density, and τ is the tortuosity. *[35, 36]* With the pore size (0.2– $1.2\ \mu m$) and permeability obtained in Figure 3, it is possible to evaluate the tortuosity of the LPS pellets at different densities: $\tau_{82\%} = 1.06$, $\tau_{89\%} = 1.34$, and $\tau_{95\%} = 1.94$. The percolating pore network becomes more tortuous as the LPS density increases, providing a longer growth path for the Li dendrite. However, the effect of this length increase (doubled from LPS-82% to LPS-95% based on the tortuosity value) on dendrite growth is much smaller than the effect of the pore size decrease, as will be discussed in the modeling section. Moreover, the Carman–Kozeny equation used here was derived with the assumption of cylindrical pores. The real pore network within the LPS pellet can be much more complicated with varying tortuosity.

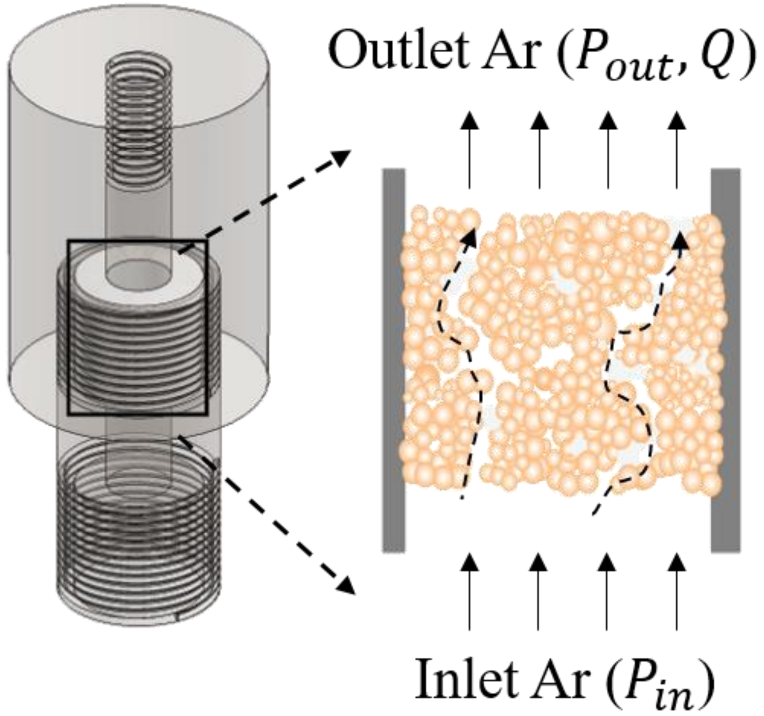

Figure SI-9. Schematic of in-house-designed apparatus for permeability test.

- **The morphology of Li filament within the pore network**

A postmortem LPS pellet in the percolating range (LPS-81.3%) is examined using the SEM to determine the lithium plating behavior inside the pellet. *Figure SI-10* shows the existence of two plating mechanisms. At first lithium is plated in the pores as depicted below, then lithium plates within the particle boundaries.

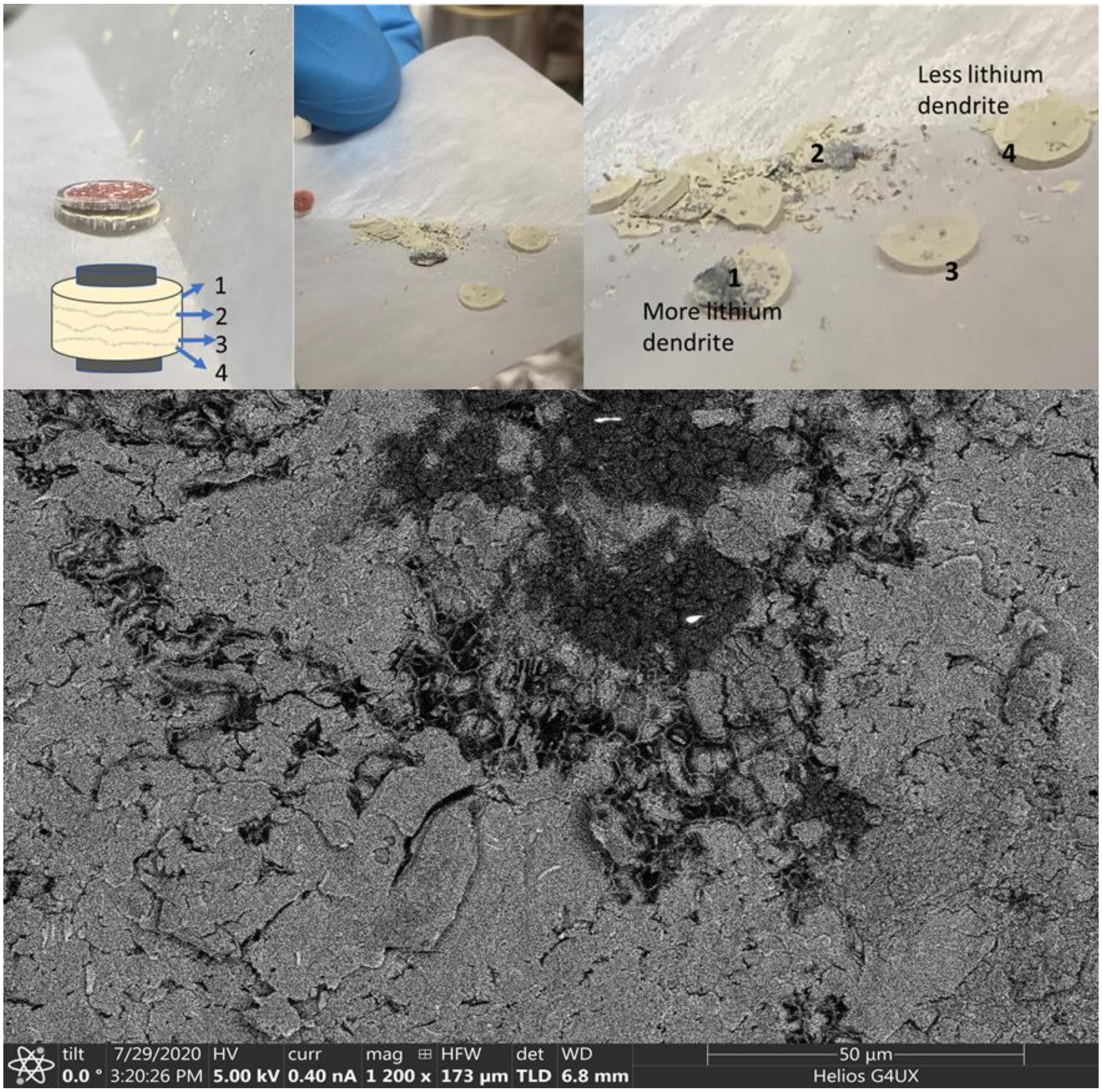

Figure SI-10. The SEM results of Li filament.

- **The distribution of overpotential and ionic current within SE**

The spatial distributions of the overpotential and the ionic current (Li+ flux) evolve in time as the dendrites propagate from the anode/SE interface into the SE. For example, the following figure shows the overpotential distribution (Figure a) and the ionic current (Figure b) when the dendrites reach a length of 10um from the Li-metal anode.

Figure a) shows a dendrite propagating into the SE from the top lithium metal electride. The overpotential is zero at the interface between the SE and Li metal anode, and at the interface between SE and Li dendrites. This value increases to 2.6 mV at a depth of 20um inside the SE. Figure b) shows the ionic current within the SE with the background color represents the overpotential. It shows that Li-ions migrate upward and deposit both on the interface at the Li metal anode and the interface at the Li dendrites.

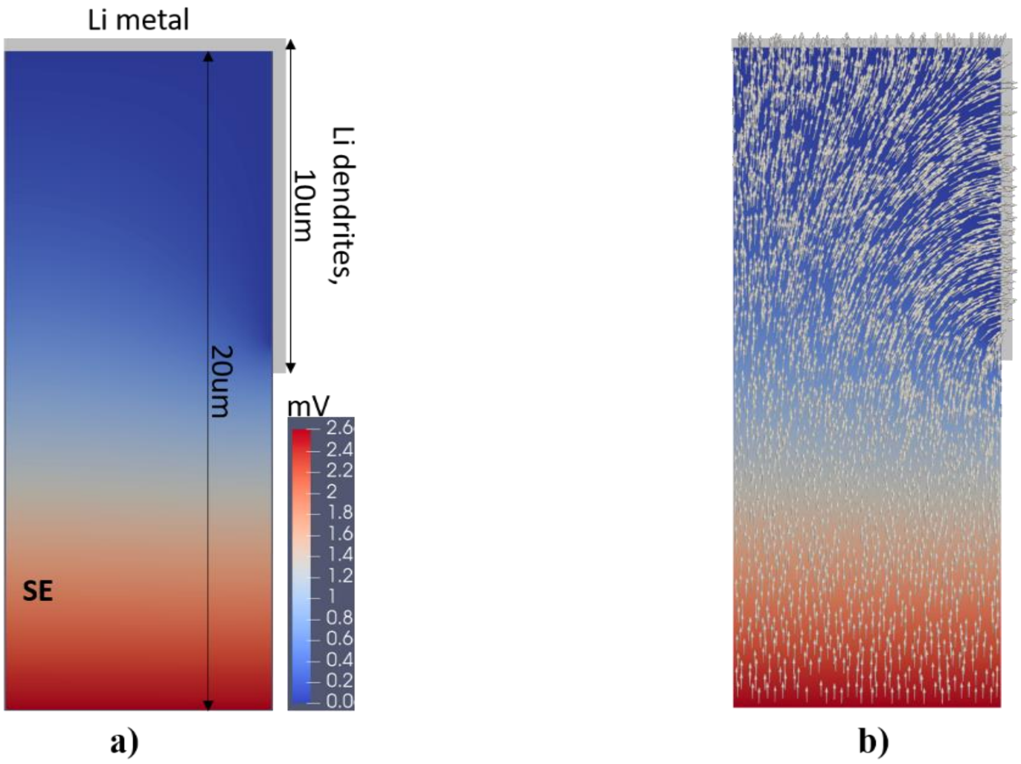

Figure SI-11. a) illustrates the overpotential distribution within the SE with unit mV, where the Li metal anode is located on the top and 10 um of Li dendrite already goes into the SE on the right top surface of the SE. Figure b) plots the corresponding ionic current in the SE

- **The effect of electronic conductivity**

It has been reported that the addition of Li_3N and LiF buffer layer (BL) between the Li metal and SE can increase the cyclability in argyrodite cells because the BL is believed to greatly reduce the electronic conductivity and neutralize the chemical reaction. This finding prompted the evaluation of our percolation hypothesis by mimicking the results presented in the work of Ji et al. [27]. Two cells were cycled at the same densities using the same cycling conditions of 0.2 mA/cm^2 for 1-h charge and discharge. Indeed, as shown in Figure SI-11, the BL increased the cyclability of the cells even in the pore-percolating region. This is because of the stable interface between the Li_3N–LiF buffer layer and Li metal. The cycling performance is related to the stable plating of Li metal, which does not react with the buffer layer, contrary to the known continuous reaction of Li metal and LPS. However, as shown in Figure SI-11 b, when the cell is tested using the long-charging criteria, the cell with the BL behaves similar to the cells without the BL. Li metal still plates inside the percolating pores, and the cell is shorted after 19 h. Although isolating chemical reactions and reducing electronic conductivity helps to increase cyclability, it does not prevent the deposition of lithium metal in percolated pores.

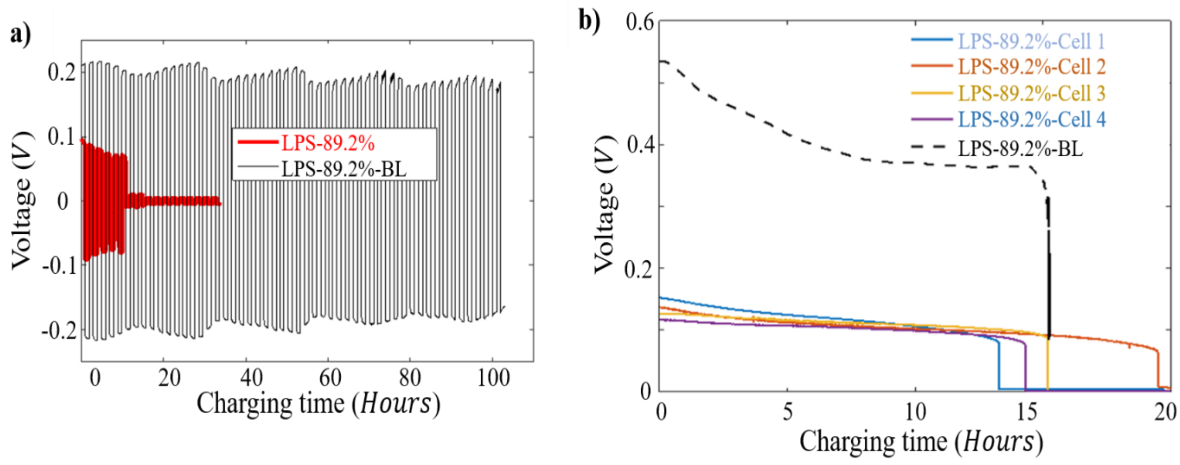

Figure SI-12. The controlling experiment by adding a buffer layer

- **The short circuit of cell with dense LPS pellet under increasing current density**

Figure SI-12 shows the cell in the non-percolating range short as current density is increased. the SE fracture should not be caused by mechanical shearing of isolated pores; however, the high hydrostatic pressure developed after Li metal fills isolated pores.

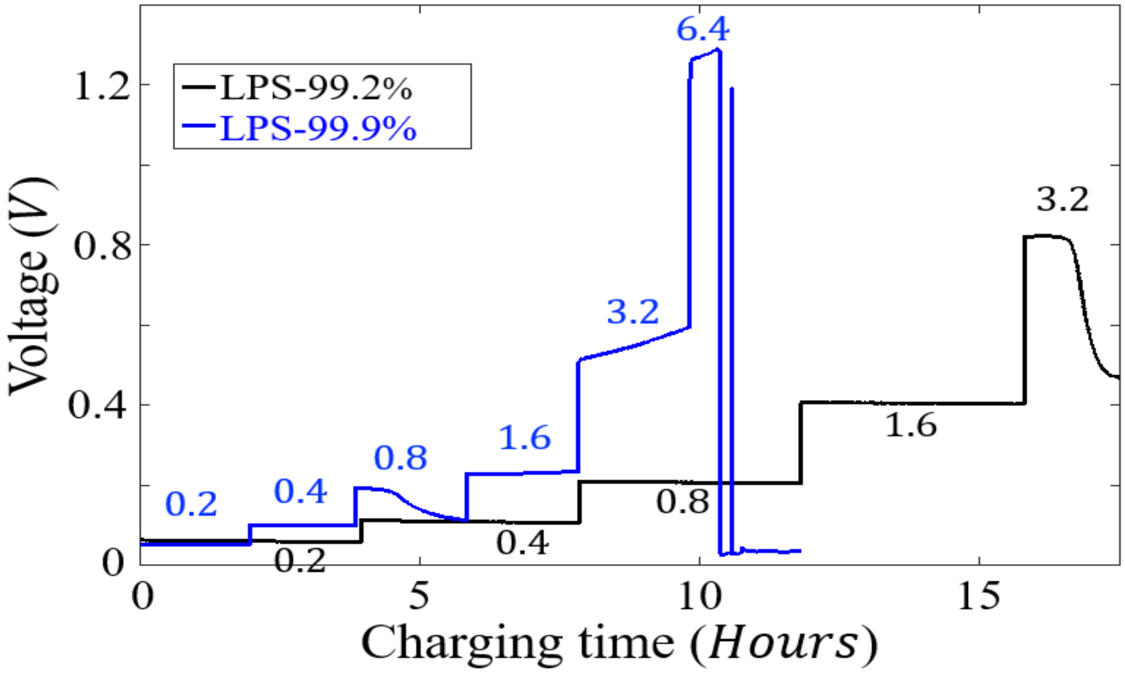

Figure SI-13. Increased current density on cells in the non-percolating regime.
